# Sexual orientation, social capital and daily tobacco smoking: a population-based study

**DOI:** 10.1186/1471-2458-14-565

**Published:** 2014-06-06

**Authors:** Martin Lindström, Jakob Axelsson, Birgit Modén, Maria Rosvall

**Affiliations:** 1 Social Medicine and Health Policy Department of Clinical Sciences, Malmö University Hospital, Lund University, Malmö S-205 02, Sweden; 2 Centre for Economic Demography, Lund University, Lund, Sweden

**Keywords:** Social capital, Sexual orientation, Tobacco smoking, Trust, Sweden

## Abstract

**Background:**

Studies have suggested poorer health in the homosexual and bisexual groups compared to heterosexuals. Tobacco smoking, which is a health-related behavior associated with psychosocial stress, may be one explanation behind such health differences. Social capital, i.e. the generalized trust in other people and social participation/social networks which decreases the costs of social interaction, has been suggested to affect health through psychosocial pathways and through norms connected with health related behaviours, The aim of this study is to investigate the association between sexual orientation and daily tobacco smoking, taking social capital into account and analyzing the attenuation of the logit after the introduction of social participation, trust and their combination in the models.

**Methods:**

In 2008 a cross-sectional public health survey was conducted in southern Sweden with a postal questionnaire with 28,198 participants aged 18–80 (55% participation rate). This study was restricted to 24,348 participants without internally missing values on all included variables. Associations between sexual orientation and tobacco smoking were analyzed with logistic regression analysis.

**Results:**

Overall, 11.9% of the men and 14.8% of the women were daily tobacco smokers. Higher and almost unaltered odds ratios of daily smoking compared to heterosexuals were observed for bisexual men and women, and for homosexual men throughout the analyses. The odds ratios of daily smoking among homosexual women were not significant. Only for the “other” sexual orientation group the odds ratios of daily smoking were reduced to not significant levels among both men and women, with a corresponding 54% attenuation of the logit in the “other” group among men and 31.5% among women after the inclusion of social participation and trust. In addition, only the “other” sexual orientation group had higher odds ratios of low participation than heterosexuals.

**Conclusions:**

Bisexual men and women and homosexual men, but not homosexual women, are daily smokers to a higher extent than heterosexuals. Only for the “other” sexual orientation group the odds ratios of daily smoking were reduced to not significant levels after adjustments for covariates including trust and social participation.

## Background

One important health policy goal is to reduce health differences between population groups in society. Health differences between population groups not only concern socioeconomic differences in health but are also defined according to gender, ethnicity, and sexual orientation. Studies in the USA [1,2], Australia [3], Europe [4] and Sweden [5] suggest poorer health in the bisexual and homosexual groups compared to heterosexuals. Discrimination, prejudice, threat of violence and violence may explain these health differences according to sexual orientation [6,7], which was also demonstrated in a previous study were we demonstrated significantly higher odds ratios of poor self rated health among bisexual, homosexual and “other” men as well as bisexual and “other” women compared to heterosexual men and women, respectively. These differences disappeared after the introduction of generalized trust in other people, experience of having been offended during the past three months, experience of threat of violence during the past twelve months and experience of violence during the past twelve months in multiple logistic regression models [7].

Differences in health related behaviours and particularly tobacco smoking prevalence may also explain health differences between population segments with different sexual orientation [8]. Most earlier studies on tobacco smoking among sexual minorities have only compared sexual minority groups with each other [9], collapsed the bisexual and homosexual groups when comparing with heterosexuals [10], or exclusively studied women [11,12]. Only one study including adults compared the homosexual and bisexual groups with heterosexuals, and found higher smoking rates for homosexual men and women and bisexual women [13]. In a UK study of adolescents aged 18–19 it was found that lesbian or gay participants were twice as likely to have a history of cigarette smoking, and bisexuals had nearly double likelilood of ever having smoked compared to heterosexual participants. Adjustment for ethnic minority status and parental socioeconomic status did not substantially alter the results. Similar results were found when combining the minority groups and comparing them with heterosexuals [14]. Despite decades of decreasing prevalence of tobacco smoking in Sweden, tobacco smoking with its relatively increasing socioeconomic gradient is still an important contributor to socioeconomic differences in health among both men and women [5]. This is due to the fact that tobacco smoking behaviours in general, as well as decisions to take up smoking or quit smoking in particular, are complex phenomena determined by psychological, economic, social and psychosocial factors [15,16], which e.g. include factors such as emotional support and instrumental support and social capital [17]. Daily smoking is thus associated not only with age, sex, country of birth [18] and socioeconomic status but also with emotional support and instrumental support [17]. Previous studies have shown sex differences in the association between sexual minority status and daily smoking (see e.g. 13), and sex differences in daily smoking exist in the general population in Sweden as well as in most other countries [19], which is the rationale for stratifying by sex in this study.

In the past fifteen to twenty years social capital has been suggested as an important health determinant, although there is still an ongoing debate concerning both the definition and the contents of the concept. Some authors define social capital as social structures, social networks, social relationships and/or institutionalized relationships [20,21]. These authors also sometimes put emphasis on the possibility for individuals to achieve their personal goals in terms of power and resources within networks by excluding trust and reciprocity from the social capital concept [20]. Other authors define social capital as social structures/relationships/networks and trust. This second group of authors also tend to put more emphasis on lowering the costs of social interaction by including trust as well as social networks in the concept [22,23]. By including both generalized trust in other people and social participation/social network in this study we theoretically adhere to the second group of authors such as Coleman and Putnam, and emphasize the lowering of social interaction costs for sexual minority groups. We thus regard social capital as mediator between sexual orientation and daily tobacco smoking. Social capital has been suggested to affect health through psychological and psychosocial pathways, through norms and attitudes connected with health related behaviours, through access to health care and amenities, and through crime [24]. Both trust and social participation have been shown to be associated with tobacco smoking [25-27], and plausible pathways connecting social capital and smoking in a causal relationship include at least the two first of the four pathways listed above. Our previous study based on the public health survey in Skåne, southern Sweden in 2008 also showed higher odds ratios of low trust in the bisexual and “other” groups compared to heterosexuals among both men and women, a pattern which may be caused by discrimination, prejudice and social exclusion [7], but this previous study did not include social participation, the other major component of social capital.

Our hypotheses are that tobacco smoking is significantly more prevalent in the sexual minority groups than among heterosexuals among both men and women, and that low social participation is significantly more prevalent in the bisexual and “other” sexual minority groups than among heterosexuals among both men and women, given the fact that we have shown that low trust is more common in these groups. The aim of this study is thus to investigate and replicate the previously found association between sexual orientation and daily tobacco smoking, and include emotional support, instrumental support, generalized trust in other people and social participation in the analyses in order to explore possible explanatory variables behind these already known associations. An additional aim is to investigate the association between sexual orientation and social participation.

## Methods

### Study population

The public health survey in 2008 regarding public health in Skåne, southern Sweden, is a cross sectional study. It is based on a random (weighted) sample of people in Skåne drawn from the public population registers. In August to September 2008, a total of 28,198 persons answered the postal questionnaire, which represents roughly a 55% response rate. Two reminder letters were also sent to initial non-respondents. In this study, the number of participants has been restricted to participants with values on all the variables included in the multiple logistic regression analyses (no internally missing values), which means that the number analysed is a total 24,348 of which 11,084 are men and 13,264 women. Ethical approval was granted by the Ethical Committee, Lund University, Sweden.

### Definitions

#### Dependent variable

*Daily tobacco smoking* was assessed by the question “Do you smoke?” which included three alternative answers “Yes, daily”, “Yes, but not daily” and “No”. In the analyses this variable was dichotomized by collapsing the two latter alternatives.

### Independent variables

*Sexual orientation* was retrieved by the item “Do you regard yourself today as 1) heterosexual, 2) bisexual, 3) homosexual, 4) other?”

*Age* was categorized into the age strata 18–24, 25–34, 35–44, 45–54, 55–64 and 65–80 years.

Stratification by *sex* was conducted in the analyses.

#### Born in Sweden/born in other country than Sweden

Participants born outside Sweden were aggregated into one group which was compared to participants born in Sweden.

*Socioeconomic status* (by occupation) included the categories employed on the labour market higher non-manual employees, medium level non-manual employees, low level non-manual employees, skilled manual workers, unskilled manual workers and self-employed and farmers. The groups outside the workforce (without occupation) consists of early retired (before age 65, for health or early retirement entitlement in the employment contract reasons), unemployed, students, old age pensioners above age 65, unclassified and long term sick leave.

*Emotional support* was measured with the item “Do you feel that you have someone or some persons who can give you proper personal support to cope with the stress and problems of life?” which had four alternatives answers: “Yes, I am absolutely certain to get such support”, “Yes, possibly”, “”Not certain”, and “No”. The three latter were collapsed as low emotional support.

*Instrumental support* was retrieved with the question “Can you get help by some or several persons in case of illness or practical problems (borrow minor items, help with reparation, help to write a letter, getting advice or information)?” which contained the same alternatives as the emotional support item, and was dichotomized accordingly.

*Generalized trust in other people* assesses the individual’s level of generalized trust in other people. It was appraised by the item “Generally, you can trust other people” which entails the four answer alternative: “Do not agree at all”, “Do not agree”, “Agree”, and “Completely agree”. These were dichotomized, the two first alternatives denoting low trust and the two latter denoting high.

*Social participation* assesses whether the respondent has taken part in the activities of formal and informal groups in society (study circle/course at workplace, other study circle/course, union meeting, meeting in other organizations, theatre/cinema, arts exhibition, church, sports event, letter to the editor of a newspaper/journal, demonstration, night club/entertainment, big gathering of relatives, private party). It is measured as an index of 13 items and dichotomized with three or less alternatives depicting low social participation, and four or more alternatives high.

### Analysis

Correlation coefficients (bivariate Pearson’s r) between emotional support, instrumental support, generalized trust in other people and social participation were calculated in order to discern psychometric independence. Prevalences (%) of daily smoking, age, birth country, socioeconomic status, emotional support, instrumental support, trust, social participation, and sexual orientation stratified by sex were assessed (Table 1). Prevalences (%) and odds ratios with 95% confidence intervals (OR:s, 95% CI) of daily smoking were calculated according to sexual orientation, age, birth country, socioeconomic status, emotional support, instrumental support, trust and social participation (Table 2). Prevalences (%), crude and age-adjusted odds ratios and 95% confidence intervals of social participation were calculated according to sexual orientation (Table 3). Age-adjusted and multiple adjusted odds ratios and 95% confidence intervals of daily tobacco smoking were calculated regarding sexual orientation. (Table 4). The attenuation of the logit for the association between the sexual orientation and daily smoking after the inclusion in the logistic regression model already containing age, country of birth and socioeconomic status (stratified for sex) of the social capital variables generalized trust in other people, social participation and their combination was calculated (not shown in Table 4). All tables were stratified by sex. The odds ratios in Tables 2, 3, 4 were calculated in logistic regression models. The statistical analyses were performed using the SPSS software package version 22.0 [28].

**Table 1:** Prevalence (%) of tobacco smoking, demographic characteristics, socioeconomic status, emotional support, instrumental support, generalized trust in other people, social participation and sexual orientation

	**Men (n = 11,084)**	**Women (n = 13,264)**	**Total (n = 24,348)**
Daily smoking	11.9	14.8	13.5
Age			
18-24	8.6	9.7	9.2
25-34	13.2	15.3	14.3
35-44	17.3	18.4	17.9
45-54	18.2	19.4	18.8
55-64	21.4	19.5	20.4
65-80	21.4	17.8	19.5
Born in other country than Sweden	12.0	12.2	12.1
Socioeconomic status (SES)			
High non-manual	11.2	9.0	10.0
Medium non-manual	13.1	18.1	15.8
Low non-manual	5.1	10.3	8.0
Skilled bluecollar	11.2	9.1	10.1
Unskilled bluecollar	11.7	11.3	11.5
Employer/farmer	8.0	3.9	5.8
Early retired	2.8	4.1	3.5
Unemployed	3.0	3.4	3.2
Student	5.1	7.0	6.1
Pensioner	23.1	18.8	20.8
No information on SES	4.8	3.5	4.1
Long term sick leave	0.8	1.3	1.1
Low emotional support	35.6	28.4	31.7
Low instrumental support	26.8	21.4	23.8
Low trust	32.8	34.4	33.7
Low social participation	40.3	35.8	37.9
Sexual orientation			
Heterosexual	96.9	97.0	97.0
Bisexual	1.1	1.5	1.3
Homosexual	0.8	0.6	0.7
Other	1.1	0.9	1.0

Men (n = 11,084), women (n = 13,264), and total (n = 24,348). The public health survey in Skåne 2008.

**Table 2:** Prevalence (%) and odds ratios (OR, 95% CI) in bivariate analyses of daily tobacco smoking according to sexual orientation, age, country of birth, socioeconomic status, emotional support, instrumental support, generalized trust in other people, and social participation

	**Men (n = 11,084)**		**Women (n = 13,264)**	
	**%**	**OR (95% CI)**	**%**	**OR (95% CI)**
**Sexual orientation**				
Heterosexual	11.7	1.00	14.6	1.00
Bisexual	22.2	2.19 (1.43-3.34)	23.6	1.81 (1.30-2.51)
Homosexual	22.4	2.18 (1.31-3.65)	11.3	0.75 (0.38-1.51)
Other	17.3	1.59 (1.00-2.12)	24.8	1.93 (1.25-2.96)
**Age**				
18-24	7.8	1.00	15.0	1.00
25-34	9.3	1.21 (0.90-1.63)	12.0	0.77 (0.63-0.95)
35-44	10.7	1.41 (1.07-1.87)	13.5	0.89 (0.73-1.08)
45-54	14.4	1.99 (1.52-2.61)	19.5	1.38 (1.15-1.65)
55-64	17.0	2.42 (1.86-3.14)	17.0	1.16 (0.97-1.40)
65-80	9.0	1.16 (0.88-1.53)	10.9	0.69 (0.57-0.85)
**Born in Sweden/born in other country than Sweden**				
Sweden	10.6	1.00	14.6	1.00
Other country	21.5	2.30 (1.99-2.66)	16.4	1.15 (0.99-1.32)
**Socioeconomic status**				
High non-manual	4.8	1.00	5.8	1.00
Medium non-manual	6.6	1.40 (1.00-1.94)	8.5	1.50 (1.13-1.98)
Low non-manual	14.1	3.23 (2.28-4.59)	16.6	3.20 (2.41-4.23)
Skilled bluecollar	13.9	3.18 (2.34-4.32)	22.1	4.57 (3.46-6.03)
Unskilled bluecollar	19.4	4.74 (3.53-6.35)	22.9	4.78 (3.65-6.26)
Employer/farmer	11.3	2.50 (1.80-3.49)	13.4	2.49 (1.76-3.53)
Early retired	31.0	8.85 (6.22-12.60)	29.9	6.87 (5.07-9.30)
Unemployed	25.8	6.87 (4.80-9.83)	23.9	5.04 (3.65-6.97)
Student	6.9	1.47 (0.97-2.23)	11.1	2.00 (1.46-2.75)
Pensioner	9.4	2.05 (1.53-2.74)	11.0	1.99 (1.52-2.61)
No information on SES	13.7	3.12 (2.18-4.47)	18.9	3.76 (2.68-5.25)
Long term sick leave	29.3	8.19 (4.88-13.75)	26.6	5.82 (3.86-8.79)
**Emotional support**				
High	10.4	1.00	13.6	1.00
Low	14.7	1.49 (1.32-1.67)	17.9	1.39 (1.25-1.54)
**Instrumental support**				
High	10.3	1.00	13,4	1.00
Low	16.3	1.68 (1.49-1.90)	19.9	1.60 (1.44-1.78)
**Trust**				
High	9.7	1.00	12.4	1.00
Low	16.5	1.84 (1.63-2.06)	19.5	1.72 (1.56-1.89)
**Social participation**				
High	8.4	1.00	11.1	1.00
Low	17.1	2.25 (2.00-2.52)	21.5	2.20 (2.00-2.43)

Men (n = 11,084) and women (n = 13,264). The public health survey in Skåne 2008.

**Table 3:** Prevalence (%), and crude and age-adjusted odds ratios (OR, 95% CI) of low social participation

	**%**	**OR (95% CI)**^**a**^	**OR (95% CI)**^**b**^
**Social parti-cipation**			
**Men**			
Heterosexual	40.0	1.00	1.00
Bisexual	43.2	1.14 (0.80-1.63)	1.36 (0.94-1.97)
Homosexual	45.9	1.27 (0.83-1.96)	1.37 (0.88-2.13)
Other	62.2	2.47 (1.72-3.55)	2.43 (1.67-3.53)
**Women**			
Heterosexual	35.7	1.00	1.00
Bisexual	29.6	0.76 (0.56-1.02)	1.07 (0.78-1.46)
Homosexual	27.8	0.70 (0.42-1.14)	0.82 (0.50-1.35)
Other	65.5	3.42 (2.31-5.04)	3.21 (2.16-4.77)

^a^Crude.

^b^Adjusted for age.

Men (N = 11,084) and women (N = 13,264). The public health survey in Skåne 2008.

**Table 4:** Age-adjusted and multiple adjusted odds ratios (OR, 95% CI) of daily tobacco smoking according to sexual orientation

**Men**				
	**OR (95% CI)**^**a**^	**OR (95% CI)**^**b**^	**OR (95% CI)**^**c**^	**OR (95% CI)**^**d**^
Heterosexual	1.00	1.00	1.00	1.00
Bisexual	2.28 (1.49-3.43)	2.07 (1.34-3.18)	2.05 (1.33-3.15)	1.95 (1.26-3.00)
Homosexual	2.21 (1.32-3.70)	2.16 (1.28-3.63)	2.14 (1.27-3.60)	2.13 (1.27-3.60)
Other	1.56 (0.98-2.48)	1.34 (0.84-2.14)	1.30 (0.81-2.08)	1.22 (0.76-1.96)
R^2^ Nagelkerke	0.006	0.026	0.027	0.032
	**OR (95% CI)**^**e**^	**OR (95% CI)**^**f**^	**OR (95% CI)**^**g**^	
Heterosexual	1.00	1.00	1.00	
Bisexual	1.95 (1.26-3.01)	1.89 (1.22-2.92)	1.88 (1.22-2.92)	
Homosexual	2.13 (1.27-3.60)	2.17 (1.28-3.67)	2.11 (1.24-3.58)	
Other	1.18 (0.73-1.89)	1.13 (0.70-1.82)	1.06 (0.66-1.72)	
R^2^ Nagelkerke	0.035	0.047	0.064	
**Women**				
	**OR (95% CI)**^**a**^	**OR (95% CI)**^**b**^	**OR (95% CI)**^**c**^	**OR (95% CI)**^**d**^
Heterosexual	1.00	1.00	1.00	1.00
Bisexual	1.83 (1.31-2.55)	1.83 (1.31-2.54)	1.71 (1.23-2.39)	1.69 (1.21-2.35)
Homosexual	0.76 (0.38-1.52)	0.74 (0.37-1.48)	0.72 (0.36-1.45)	0.70 (0.35-1.41)
Other	1.92 (1.25-2.95)	1.88 (1.22-2.90)	1.78 (1.16-2.75)	1.70 (1.11-2.63)
R^2^ Nagelkerke	0.003	0.003	0.006	0.010
	**OR (95% CI)**^**e**^	**OR (95% CI)**^**f**^	**OR (95% CI)**^**g**^	
Heterosexual	1.00	1.00	1.00	
Bisexual	1.65 (1.18-2.30)	1.59 (1.14-2.22)	1.68 (1.20-2.36)	
Homosexual	0.70 (0.34-1.40)	0.71 (0.35-1.43)	0.76 (0.38-1.54)	
Other	1.66 (1.08-2.56)	1.59 (1.03-2.46)	1.44 (0.93-2.23)	
R^2^ Nagelkerke	0.014	0.024	0.048	

^a^Adjusted for age.

^b^Adjusted for age and country of origin.

^c^Adjusted for age, country of origin and socioeconomic status.

^d^Adjusted for age, country of origin, socioeconomic status and emotional support.

^e^Adjusted for age, country of origin, socioeconomic status, emotional support and instrumental support.

^f^Adjusted for age, country of origin, socioeconomic status, emotional support, instrumental support and generalized trust in other people.

^g^Adjusted for age, country of origin, socioeconomic status, emotional support, instrumental support, generalized trust in other people, and social participation.

Men (N = 11,084) and women (N = 13,264). The public health survey in Skåne 2008.

## Results

All correlations between the social support and social capital variables were low, with the exception of the correlation coefficient (bivariate Pearson’s r) between emotional support and instrumental support (r = 0,568). The correlation coefficient between emotional support and trust was 0.128, between emotional support and social participation 0.154, between instrumental support and trust 0.143, between instrumental support and social participation 0.179 and between trust and social participation 0.137.

Table 1 shows that 11.9% of the men and 14.8% of the women were daily tobacco smokers. The distribution (prevalence) for age, country of birth, socioeconomic status, emotional support, instrumental support, trust, social participation and sexual orientation are also displayed (Table 1).

Table 2 demonstrates that the odds ratios and prevalence (%) of daily tobacco smoking in bivariate analyses were significantly higher among middle-aged respondents, respondents with lower socioeconomic status, low emotional support, low instrumental support, low trust, low social participation and among persons of bisexual and other orientation among both men and women. The group men born abroad had a higher odds ratio of daily smoking than men born in Sweden, and homosexual men also had higher odds ratios of daily smoking compared to heterosexual men.

The crude and age-adjusted odds ratios in Table 3 display that only the “other” sexual orientation group had a significantly higher prevalence of low social participation compared to the heterosexual reference group. In the age-adjusted models, the odds ratio of low social participation in the “other” sexual orientation group was 2.43 (1.67-3.53) among men and 3.21 (2.16-4.77) among women compared to the heterosexual reference group.

The higher odds ratios of daily smoking among bisexual and homosexual men compared to heterosexual men remained throughout the multiple logistic regression analyses. In the final analysis the odds ratios of daily smoking were 1.88 (1.22-2.92) among bisexual and 2.11 (1.24-3.58) among homosexual men. In contrast, the odds ratio of daily smoking became not significant already in the second model for the “other” sexual orientation category among men. Among women, the odds ratios of daily smoking for the bisexual group were also higher and almost unaltered throughout the analyses, odds ratio 1.68 (1.20-2.36) in the final model. In contrast, no statistically significant differences between homosexual and heterosexual women were observed throughout the multiple analyses, odds ratio 0.76 (0.38-1.54) in the final model. The odds ratios of daily smoking for the “other” sexual orientation category among women were significant until social participation was added in the final model, an addition which reduced the odds ratio of daily smoking in this group from 1.59 (1.03-2.46) to 1.44 (0.93-2.23). When social participation, trust and their combination were added to the logistic regression model assessing the association between sexual orientation and daily smoking including age, country of birth and socioeconomic status (stratified for sex), only the attenuation of the logit for the “other” sexual orientation category was substantial (above 10%) for both men and women, 20.8% for trust, 40.4% for social participation and 54.2% for their combination among men and 10.6% for trust, 26.5% for social participation and 31.5% for their combination among women. A substantial attenuation of the logit was also observed for homosexual women when social participation (21.0% attenuation) and the combination of trust and social participation (25.5% attenuation) were added to the logistic regression model including age, country of birth and socioeconomic status.

## Discussion

Major social capital components such as trust and social participation do not reduce the significantly higher odds ratios of daily smoking in the sexual minority groups, with the exception of the inclusion of social participation in the final model for the “other” group among women. Also, the addition of trust, social participation and their combination to the logistic regression model already including age, country of birth and socioeconomic status substantially reduced the logit for the association between sexual orientation and daily smoking in the “other” sexual orientation group. A substantial attenuation of the logit was also observed for homosexual women when social participation and the combination of social participation and trust were added to the logistic regression model already including age, country of birth and socioeconomic status. No substantial attenuation of the logit (less than 10%) was observed for homosexual and bisexual men and bisexual women. One reason is that there seem to be no significant differences in social participation according to sexual orientation for bisexual and homosexual men and women, which is the second finding of our study.

Bisexual men and women have significantly higher odds ratios of daily smoking throughout the analyses compared to heterosexual men and women, respectively, and the odds ratios remain almost unaltered even after the inclusion of the two social capital variables. The two social capital components trust and social participation can thus not account for the high smoking prevalence in this sexual minority group. In sharp contrast, there are distinct differences between the comparisons of the odds ratios of daily smoking between homosexual and heterosexual men as opposed to the corresponding comparison between homosexual and heterosexual women. Significantly higher odds ratios of daily smoking remain among homosexual men throughout the analyses, while no significant odds ratios among homosexual women compared to heterosexual women are observed. Only the odds ratios of daily smoking for the “other” sexual orientation group become not statistically significant in the analyses, for men already after inclusion of birth country and for women after inclusion of social participation. (Table 4). Finally, only the “other” sexual orientation group has higher odds ratios of low social participation among both men and women.

The higher odds ratios of daily smoking among bisexual men and women partly correspond with the finding that bisexual women but not men had statistically increased risk of smoking compared to female and male heterosexuals, respectively, in a study from the UK. The finding of that study also conforms with our finding that homosexual men had increased odds of daily smoking compared to heterosexual men, although homosexual women did not have increased odds of daily smoking in our study in opposition to the finding of the UK study that homosexual women had increased risk of being daily smokers [13]. On the other hand, one study which exclusively concerns women suggests that homosexual women have a lower risk of daily smoking than heterosexual women [12]. Given the small proportion of sexual minorities in most studies, one interpretation is that partly different findings may be explained by methodological concerns such as e.g. selection bias. However, a second more plausible interpretation is that sexual minorities live in different social settings which may explain the observed differences. Such patterns may most probably include various aspects of discrimination, i.e. “the dislike of the unlike” [29]. This second interpretation seems to be the most likely, given the fact that our results correspond well with a Swedish government investigation published in 2005 which showed that homosexual and bisexual men were overrepresented as daily smokers compared to heterosexual men, while the differences in daily smoking between homosexual and bisexual women compared to heterosexual women were smaller [30]. In addition, the finding that the “other” group has significantly lower social participation may be regarded as an aspect of what has sometimes been called the exclusive “dark side of social capital” [31]. Since most daily smokers are recruited during adolescence and in early adulthood [32], it seems that one preventive strategy would be to stop recruitment of daily smokers in these minority groups during adolescence and early adulthood.

By including both generalized trust in other people and social participation in this study we theoretically and conceptually adhere to the group of authors such as Coleman and Putnam, and thus emphasize the lowering of social interaction costs for sexual minority groups rather than the individual’s struggle for resources within networks. On the other hand, the authors who only acknowledge social networks and not trust as the core component of social capital have had problems operationalizing the struggle for power and resources within the social networks [33]. Our social participation variable is similar to those network variables used by authors within the literature that defines social capital exclusively as social networks. It should be noted that our trust variable conceptually falls within the social capital literature tradition including Coleman and Putnam [22,23] as an aspect of social capital and not primarily an individual trait. Vast differences in trust prevalence (“Do you trust others”) between different countries indicate the social and societal as opposed to the individual aspect of generalized trust in other people [34].

Men born abroad have a significantly higher odds ratio of daily smoking compared to men born in Sweden, while the odds ratio of daily smoking among women born abroad is not significantly higher than among women born in Sweden. These patterns have been previously explored, and the results indicate that men born in most other countries than Sweden have higher odds ratios of daily smoking, while women born abroad show differing patterns with high odds ratios of daily smoking for women born in e.g. Denmark but low odds ratios for women born in e.g. Arabic speaking countries compared to women born in Sweden [18].

### Strengths and limitations

The 55% participation rate may theoretically be a source of selection bias, but a previous study on an earlier similar questionnaire with a similar response rate in Skåne showed a good correspondence with population registers concerning composition of the population according to age, gender, education and socioeconomic status, with the exception of under-representation observed among people born in other countries than Sweden [35]. Calculations on the 2008 public health survey in Skåne also display under-representation in the age group 18–34 years (22.0% among respondents but 29.0% in the original sample), and a corresponding over-representation in the 65–80 year age group (22.9% of respondents compared to 18.0% in the sample). Some extent of under-representation of men (45.1% among respondents and 50.0% in the sample) was also observed. People with low education were also under-represented to some extent (25.2% among respondents and 29.3% in the original sample). However, the more important under-representation among respondents was observed among people born outside Europe (4.1% among respondents but 6.9% in the sample) [36]. The risk of selection bias may still be regarded as acceptably low.

The low proportion of the population which belongs to sexual minorities corresponds well with other national level Swedish data [37] and data from the USA [10]. Still, there may be limitations with items focusing on identity at a given point in time which may plausibly result in under-representation to some extent due to misclassification as a result of some remaining social desirability bias. Also, the fact that aspects of sexual orientation other than identity, e.g. attraction and behavior, were not included in the survey may also be regarded as a limitation [14].

The potential confounders age, birth country, socioeconomic status and social support as well as trust and social participation were adjusted for, and stratification according to sex was conducted.

The tobacco smoking items are valid and reliable for the assessment of tobacco smoking in population studies [38,39]. The item concerning sexual orientation has been used previously in a study conducted by a Swedish state authority [37]. The low correlation coefficients between the social support and social capital variables indicate that the variables measure separate dimensions of social support and social capital. The only strong r = 0.568 correlation between emotional and instrumental support indicates correlation that is high but not high enough to indicate the same dimension of social support.

The cross-sectional study design makes all conclusions involving causation formally impossible.

## Conclusions

Higher and almost unaltered odds ratios of daily smoking compared to heterosexuals are observed for bisexual men and women, as well as for homosexual men throughout the multiple analyses. In contrast, the odds ratios of daily smoking among homosexual women do not significantly differ. Only for the “other” sexual orientation group the odds ratios of daily smoking are reduced to not significant levels compared to heterosexuals among both men and women. Only the “other” sexual orientation group has higher odds ratios of low participation among both men and women compared to heterosexuals.

## Competing interests

The authors declare that they have no competing interests.

## Authors’ contributions

ML and MR has contributed to the conception and drafting of the work. ML has analysed the data and written the first draft of the manuscript. ML, JA, BM and MR have contributed to the interpretation and the discussion of the results, and the revision of the contents. All authors have read and approved the final manuscript.

## Pre-publication history

The pre-publication history for this paper can be accessed here:

http://www.biomedcentral.com/1471-2458/14/565/prepub
